# Seasonal changes of the diurnal variation of precipitation in the upper Río Chagres basin, Panamá

**DOI:** 10.1371/journal.pone.0224662

**Published:** 2019-12-16

**Authors:** Tosiyuki Nakaegawa, Reinhardt Pinzon, Jose Fabrega, Johnny A. Cuevas, Hector A. De Lima, Eric Cordoba, Keisuke Nakayama, Josue Ivan Batista Lao, Alcely Lau Melo, Diego Arturo Gonzalez, Shoji Kusunoki

**Affiliations:** 1 Department of Applied Meteorology Research, Meteorological Research Institute, Tsukuba, Ibaraki, Japan; 2 Centro de Investigaciones Hidráulicas e Hidrotécnicas, Universidad Tecnológica de Panamá, Panamá, Panamá; 3 Sistema Nacional de Investigación, Secretaria Nacional de Ciencia y Tecnología e Innovación, Panamá, Panamá; 4 Sección de Recursos Hídricos, Autoridad del Canal Panamá, Panamá, Panamá; 5 Department of Civil Engineering, Kobe University, Kobe, Japan; 6 Instituto Profesional y Técnico de Capira, Capira, Panamá; 7 Hydrometeorological Departnment, Empresa de Transmisión Eléctrica S.A., Panamá, Panamá; 8 Department of Earth System Modeling Research, Meteorological Research Institute, Tsukuba, Ibaraki, Japan; Universidade de Vigo, SPAIN

## Abstract

This study elucidated the characteristics of climatological seasonal changes in the diurnal variations of precipitation at four ground stations in the upper Río Chagres basin in the Panama Canal watershed. The seasonal changes differed among the stations, although they are located within an area of only 414 km^2^. Precipitation peaks in the early afternoon at 1500 local standard time (LST) were observed at all the stations. At Chamon, monthly-mean hourly precipitation at every hour exceeded 0.3 mm h^–1^ throughout November and December. The occurrence of morning precipitation in January and March distinguished the seasonal precipitation pattern at Esperanza from the pattern at the other stations. Analyses of the seasonal changes in the diurnal variation with pattern correlations and rotational empirical orthogonal functions grouped the stations into two pairs: no morning peak at Chico and Río Piedras in the downstream basin and morning peak at Chamon and Esperanza in the upstream basin.

## Introduction

Both diurnal and seasonal variations of precipitation influence land-surface hydrometeorological processes and, thus, biosphere, including human beings that use water and solar energy. Hydrometeorological processes that display diurnal variations include convective cloudiness [1], the atmospheric hydrologic cycle [2], streamflow [3], the groundwater table [4], sediment flow [5], and soil respiration [6]. Diurnal variations of precipitation are a primary driver of these hydrometeorological diurnal variations.

Panama is located in between the Caribbean Sea and Pacific Ocean and climatologically affected by both which bring high humidity (e.g. [7, 8]). Panama has two seasons, a dry season and a rainy season [9] with different local onset and withdrawal dates [10]. The migration of the Inter-Tropical Convergence Zone (ITCZ) primarily induces this seasonal march [11, 12]. Strong El Niño Southern Oscillation events distinctly affect Panama: higher than normal precipitation and air temperature during strong El Niño events [13], and lower than normal precipitation and air temperature during strong La Niña events [14]. There are two major large-scale weather systems, disturbances originated from the eastern Pacific ITCZ in September and June [14], and cold surges penetrated from northern high latitude to southern Panama in November-December-January [15]. These weather systems trigger convective activities and bring precipitation to the Pacific Coast and the Caribbean Coast, respectively.

The convective activities have strong diurnal variations with the diurnal variations of precipitation, induced by the diurnal variation of solar energy. Diurnal variations of precipitation have been investigated in many parts of the world, such as Colombia [16], Costa Rica [17], and Malaysia and Japan [18]. However, in Central America, diurnal variations of precipitation based on ground observations have rarely been explored primarily owing to a lack of precipitation data with a sufficiently high temporal resolution. There are five major patterns of diurnal and semidiurnal variations of precipitation in Colombia, including a precipitation minimum during the morning hours, and local features that differed among stations [16]. A precipitation peak in the early afternoon in central Costa Rica was reported at a site far from both coasts that was related to the diurnal variation of storm precipitation [19]. Although diurnal precipitation variations have not yet been investigated in Panama by using ground observations, they have been investigated by using satellite observations (e.g. [20, 21]). A morning precipitation peak is induced in the Gulf of Panama by a land breeze along the highly convergent wind coastline [22]. A complex diurnal variation in the Isthmus of Panama, and a strong early afternoon peak over land from 1400 to 1800 local standard time (LST; = UTC –5 h) over land was reported [23]. There are three tropical diurnal precipitation variation regimes globally [24]; they classified the diurnal variation in Central America, including Panama, as belonging to a landside coastal regime with precipitation peaks occurring from noon to evening.

**Fig 1:**
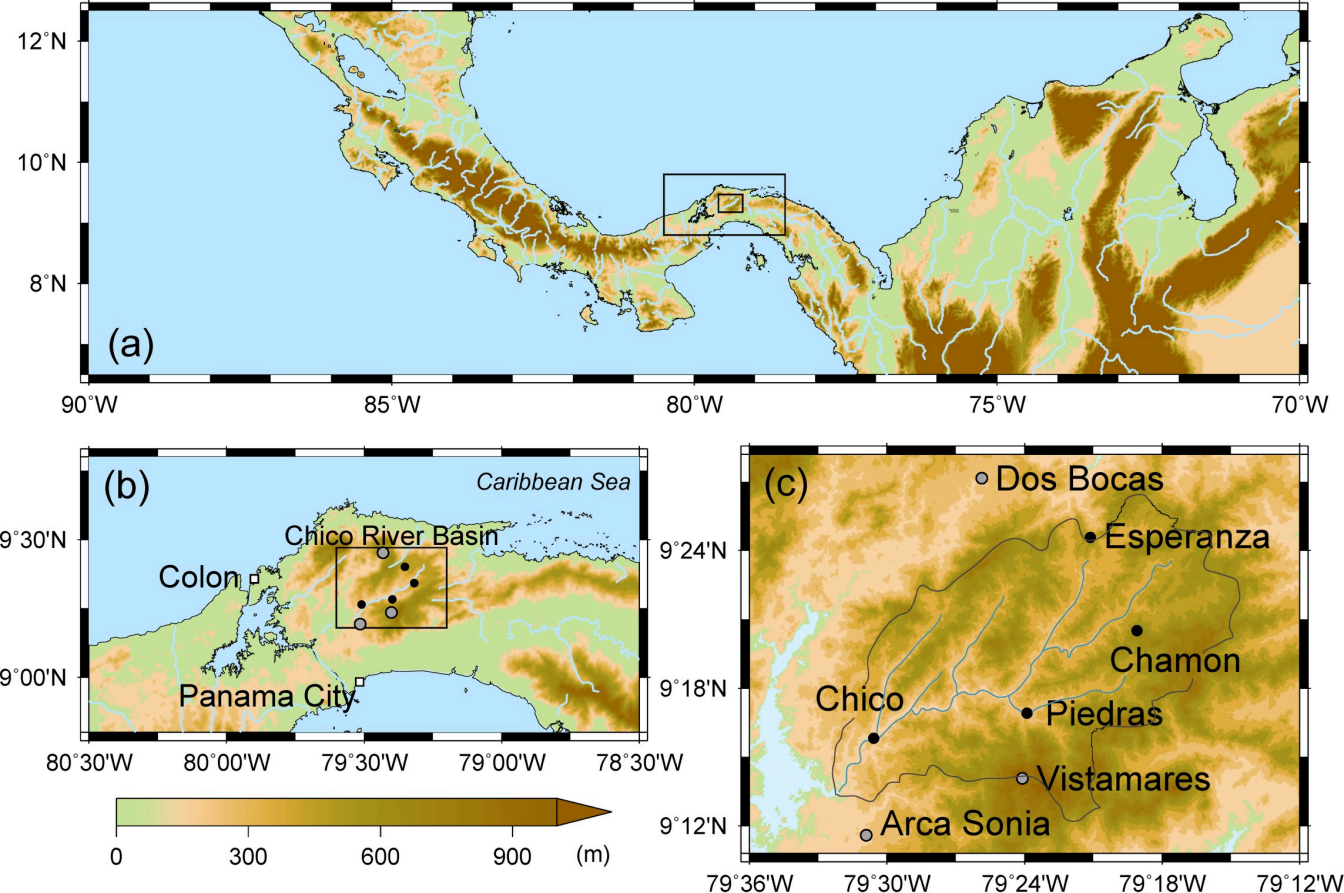
(a) Large scale geographical map with the target river basin centered. (b) Location map of the upper Rio Chagres basin, Panama. (c) Locations of the four rain gauge stations in the upper Rio Chagres basin. The boxes in Fig 1A show the area included in b and c. The box in Fig 1b shows the area included in Fig 1c. Light blue represents seas in Fig 1B and Lago Alhajuela in Fig 1C. SRTM 3-arc-second or 90 m Digital Elevation Data was used to draw these maps (doi: 10.5066/F7F76B1X).

Orography also affects the diurnal variations because it induces atmospheric circulations with orographic uplift of moist air [16] and katabatic-anabatic mountain winds. Seasonal changes of partitioning of net radiation at the Earth’s surface to sensible and latent heats affects the diurnal variation of precipitation through atmospheric stability [25]. On interannual time scale, El Niño–Southern Oscillation (ENSO) [16, 26] and Madden-Julian Oscillation (MJO) affect the diurnal variation by changing the amplitude but not the phase [16, 27]. The upper Río Chagres basin is a sub-basin on the eastern side of the Panama Canal watershed (Fig 1). The hydrology, ecology, and geology of this basin have been investigated in detail because it lies in one of the most important and complex tropical rainforest regions of the world [28]. A single precipitation peak at 1500 LST was identified with ground observations to calculate the annual mean diurnal variation of precipitation for the entire sub-basin and identified [29], but the geographical and seasonal distributions of the diurnal variation have not yet been explored. Another feature of this mountainous basin can modulate convection activities [30–32], and affect the diurnal variation of precipitation.

The aim of this study was to characterize climatological seasonal changes of the diurnal variation of precipitation at four ground stations in the upper Río Chagres basin. We analyzed the hourly precipitation pattern in each month of the year and examined how the characteristics of the diurnal variation differed over the course of the year and among the four stations.

### Study area

The four rain gauge stations are located in the upper Río Chagres basin (Fig 1B), which contains five major streams: Río Chico, Río Limpio, Río Esperanza, Río Chagrecito, and Río Piedras. The basin area of 414 km^2^ accounts for 12% of the entire Panama Canal watershed. The elevation ranges from 60 m to 1000 m, and more than 37% of the basin is occupied by steep hillsides with gradients greater than 45°. The entire basin lies within the Chagres National Park, which was established in 1985 to preserve the drinking water source of Panama City and Colon and the flow of water into the canal [33]. The dominant vegetation type in the upper Río Chagres basin is tropical rainforest. The rainy season in Panama starts in May and ends in December [9], but its timing varies in different parts of the basin [10].

## Methods and data

### Methods

We computed climatological mean precipitation (*DP*(*m*,*h*)) for each hour of day and for each month from hourly precipitation data (*P*(*y*,*m*,*h*)),

$$DP\left(m,h\right)=\frac{1}{N}\sum_{y=1}^{N}P\left(y,m,h\right)$$ (1)

where *y*, *m*, and *h*, represent year, month, and hour (LST), and *N* is the number of years used in the analysis. Similarly, we calculated the climatological mean precipitation separately for each month (*MP*(*m*)) and (*HP*(*h*)) each hour of day as

$$MP\left(m\right)=\frac{1}{24}\sum_{h=1}^{24}DP\left(m,h\right)$$ (2)

$$HP\left(h\right)=\frac{1}{12}\sum_{m=1}^{12}DP\left(m,h\right)$$ (3)

We do not apply the normalization to the diurnal variation analysis following previous works [23,24] since the precipitation amount is important information for climate service.

We applied a rotated empirical orthogonal function (REOF) analysis to the correlation matrix of the seasonal changes of the diurnal variation of precipitation at the four stations, as described in [24]. The precipitation were standardlized with climatological annual mean precipitation before applying the REOF analysis.

### Data

Hourly precipitation data were analyzed at four rain gauge stations: Chico, Río Piedras, Chamon, and Esperanza (Fig 1 and Table 1). These data were operationally observed by the Autoridad del Canal Panama. Chico station is located just upstream from where Río Chagres enters Lago Alhajuela. Río Piedras as rain gauge station is located near the confluence of Río Chagres and Río Piedras. Chamon is located on a northwestern slope in the headwaters region of the mainstream of the Río Chagres. Esperanza is on the divide forming the northern boundary of the basin above the headwaters of Río Esperanza. Observation data from Chico and Río Piedras are available for more than 40 years, since the early 1970s, and observations have been recorded at Chamon and Esperanza for more than 17 years, since around 2000. For this analysis, we used data from the 17-year period from 2000 to 2016, which is hereafter referred to as the climatological mean. This time span is short compared to the standard span of climatology to average interannual variabilities of meteorological variables; however it is meaningful to analyze the diurnal variation since there has not yet been a study on this aspect. In addition to the four stations, we analyzed hourly precipitation data at three station adjacent to the Upper Río Chagres basin: Vistamares close to the southern watershed of the basin, Dos Bocas on the Caribbean Sea side, and Arca Sonia on Pacific Ocean side.

**Table 1:** Station locations and elevations, and climatological annual mean precipitation at each of the four rain gauge stations in the upper Rio Chagres basin.

Station name	Longitude (W)	Latitude (N)	Elevation (m)	Annual Precipitation (mm year^–1^)	period
Chico	79° 30' 35"	9° 15' 49"	104	2820	2000–2016
Río Piedras	79° 23' 52"	9° 16' 52"	198	2903	2000–2011
	79° 23' 58"	9° 16' 55"	201	2514	2011–2016
Chamon	79° 19' 06"	9° 20' 31"	2100	3782	2000–2016
Esperanza	79° 21' 08"	9° 24' 35"	1780	5058	2000–2016
Vistamares	79° 24' 05"	9° 14' 04"	969	2733	2000–2016
Dos Bocas	79° 25' 52"	9° 27' 09"	250	4792	2000–2016
Arca Sonia	79° 30' 54"	9° 11' 36"	261	2759	2000–2016

The rain gauges used in these stations are tipping bucket type one (Model 5050P; HydroLynx Systems, Inc.). The tipping volume is 1 mm and the accuracy is ±3% for 0 to 50 mm h^-1^. The operational maintenance is performed by the Autoridad del Canal Panama at least 3 times per year. Quality checks of the data are essential before an analysis [34]. The raw 5-min data are quality controlled with a commercial software (AQUARIUS Time-Series, version 3.7; https://aquaticinformatics.com/; Copyright, 2019 Aquatic Informatics Inc.) which has a portfolio of features for real-time sanity checking, error detection, data cleaning, data flagging, automatic bias corrections, and rating shift management, and hourly data are derived. We checked the homogeneity of the data with a free software RHTestV4 [35, 36, 37] (http://etccdi.pacificclimate.org/software.shtml, freely available on request, Copyright, Environment Canada, 2012, GNU Lesser General Public License) after constructing daily data from the hourly data and found no obvious inhomogeneity in the data.

The station at Rio Piedras was relocated about 200 m away from the original site to new one and the operation at the new site has started since 2011. Then, we analyzed the time series of hourly precipitation intensity for 2011 to 2014 when the observations were conducted at the two sites. The temporal correlation between them is high with the correlation coefficient of 0.98 and the interception is 1.44 mm day^–1^, indicating precipitation at the original site is higher than that of the new site. Annual mean precipitation between the two sites in Table 1 is not statistically different at 5% significance level with Wetch’s test due to large interannual variabilities influenced by ENSO and others. This result allows us to use a continuous time series of precipitation at a virtual single station by connect the two time series of the two sites.

We used three threshold precipitation intensity values to define the onset and withdrawal dates of the rainy season: 3 and 5 mm day^–1^, and the climatological annual mean precipitation intensity. The threshold value of 3 and 5 mm day^-1^ are based on previous works [38, 39] respectively; that of climatological annual mean is based on Indian Meteorological Department [40]. We applied them here to monthly mean precipitation to be consistent with the monthly-timescale analysis of the diurnal variations in this study.

We also used six-hourly wind data for the grid point nearest to the Upper Río Chagres basin (located at 9.25˚N, 79.25˚W) obtained from the European Centre for Medium-range Weather Forecast Reanalysis dataset (ERA5; [41]) to examine seasonal changes in climatological diurnal large-scale near-surface wind patterns. The horizontal resolution of these data is about 31 km. The elevation of this grid (202 m) in the atmospheric general circulation model used for ERA5 is lower than that of all of the observation stations except Chico. Wind data for one of the lowest atmospheric level of ERA5, 100 m from the ground, were used in the analysis to represent land and sea breezes. Katabatic-anabatic mountain winds can produce the low-level convergence, increase convections, and modulate the diurnal variations of precipitation; however, no wind data which can resolve katabatic-anabatic mountain winds is available. We used the same analysis period for the wind data as for the precipitation data. The monthly diurnal variation of the wind is computed by subtracting the climatological monthly mean. The annual mean diurnal variation of the wind is computed in the same way as in the monthly one but by subtracting the climatological annual mean. Monthly mean wind anomaly is computed as in the annual mean diurnal variation.

Ground hourly wind is available only at Corozal Oeste (79° 34' 29"W, 8°58' 50"N, 7 m a.s.l.) and Limon Bay (79° 54' 54"W, 9° 21' 19"W, 3 m a.s.l.), although these weather stations are outside the Upper Chagres River basin. Limon Bay is located on a pier next to the water of the Gatun Lake, the Panama Canal reservoir on the Caribbean Sea side, whereas Corozal Oeste is about 5 km inland from the Pacific Ocean side. The same analysis of the ERA5 wind is applied to the ground hourly data.

We examine modulation of ENSO to the diurnal variation of precipitation on annual mean time-scale but not a monthly one during El Niño and La Niña years. ENSO years were obtained from NINO.3 Sea Surface Temperature (SST) index, normalized area averaged SST from 5°S-5°N and 150°W-90°W (https://www.data.jma.go.jp/gmd/cpd/data/elnino/index/nino3idx.html) and the multivariate ENSO index (https://www.esrl.noaa.gov/psd/enso/mei/); 2002/03, 2009/10, and 2015/2016 for El Niño, and 2000/2001, 2007/2008, 2010/2011, and 2011/2012 for La Niña. An ENSO year is defined as April of year 0 during an onset through March of year +1 or next year by taking account of a typical ENSO years and hydrological year.

Hourly streamflow of the Upper Chagres River at Chico at Chico is used to examine whether or not it has a diurnal variation. The data were used for a 9-year period from 2000 to 2009 due to our availability.

### Climatological annual precipitation and the rainy season

The climatological annual mean precipitation at each station (Table 1) exceeded 2730 mm year^–1^ at all stations; this value is about four times the global land area mean of around 700 mm year^–1^ and close to the mean of 2715 mm year^–1^ for Panama as a whole. Chico, Río Piedras, Arca Sonia, and Vistamares, which are in the downstream part of the upper Río Chagres basin, had similar means such as 2733 to 2820 mm year^–1^. Chamon, which is near the eastern edge of the basin, received nearly 3800 mm year^–1^, and Esperanza, on its northern edge, received more than 5000 mm year^–1^. Dos Bocas to its north of the basin has similar amount. Orographic precipitation is generally dependent on elevation (e.g. [8, 42]), but the climatological annual mean at these stations did not show a simple relationship with station elevation. Therefore, the complicated topography of the basin apparently affects the climatological mean annual precipitation amounts at each station.

The onset of the rainy season in the upper Río Chagres basin occurred in April or May, depending on the threshold value used (Table 2; see the Data subsection). It is noteworthy that at Esperanza, monthly-mean precipitation exceeded the threshold value of 5 mm day^–1^ year-round, and at Chamon it exceeded the threshold value of 3 mm day^–1^ year-round. At Chico, the rainy season ended in December, and at Río Piedras and Chamon, it ended in January. We attribute the geographical variation in the onset and withdrawal dates of the rainy season in this small basin primarily to the variation in the climatological mean annual precipitation.

**Table 2:** Onset and withdrawal dates of the rainy season at each station in the upper Rio Chagres basin according to each of the three threshold values.

	Onset			Withdrawal		
Station name	3 mm day^−1^	5 mm day^−1^	Annual mean	3 mm day^−1^	5 mm day^−1^	Annual mean
Chico	April	May		December		
Río Piedras	April	May		January		
Chamon	-	April	May	-	January	
Esperanza	-		May	-		December

Climatological monthly precipitation shows different features of number and phase of the peak among the stations such as local peaks in August and November at Chico, in June and October at Rio Piedras, Chamon in November, and Esperanza; these differences in phases may be due to local-scale complex orographic convection activities induced in the ITCZ migration and intraseasonal oscillations that module the convective activity in the tropics as MJO [27].

## Results

**Fig 2:**
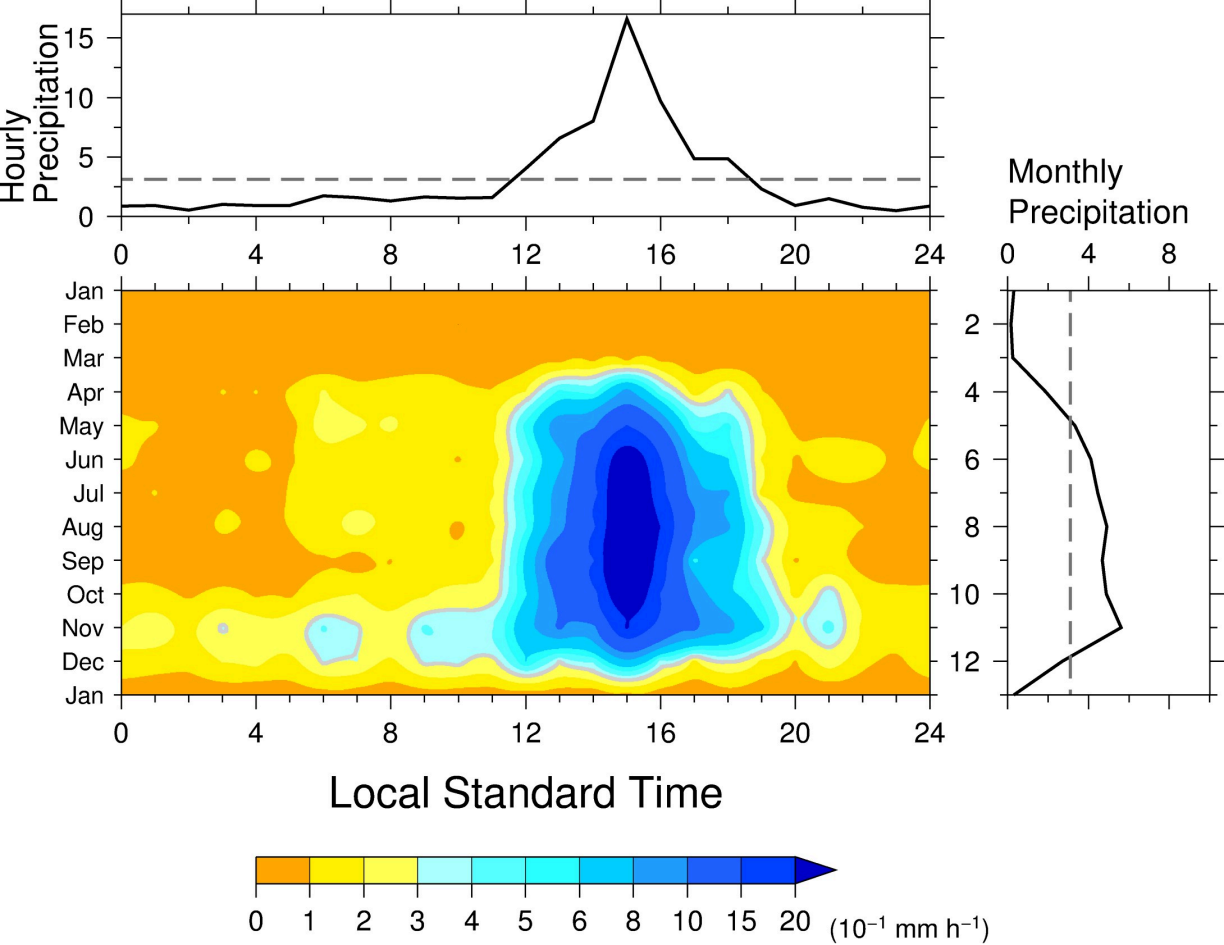
(center) The diurnal variation of precipitation during the year at Chico station. (right) Monthly mean precipitation. (top) Annual mean diurnal variation of hourly precipitation. The gray contours in (center) denote annual mean precipitation. The dashed lines indicate the annual mean hourly (top) and monthly (right) precipitation.

At Chico, precipitation showed a distinct diurnal variation from April to December with a precipitation peak in the afternoon (Fig 2, center panel), when the precipitation intensity exceeded the climatological annual mean. The precipitation intensity exceeded 1 mm h^−1^ from 1300 to 1700 LST from May to November, consistent with the landside coastal regime in a satellite-based study [24]. From January to March, precipitation was weak throughout the day, and even from April to December, precipitation weakened in the morning. This contrast between morning and afternoon characterized the diurnal variation from May to November. The climatological annual mean hourly precipitation also showed a distinct diurnal variation with a clear peak at 1500 LST (Fig 2, top panel), when hourly precipitation from May to November exceeded 1 mm h^−1^. The timing of this peak is the same as that obtained by previous study [28] for the sub-basin mean. The climatological monthly precipitation showed distinct seasonal changes characterized by two local peaks (Fig 2, right panel). The northward and southward ITCZ migration forms a single dominant peak or rainy and dry seasons [9,10]. Local minimum in September is out of phase of mid-summer drought during July and August [14]. Similar seasonal march is found in San Pedro close to Colon and Cocle de Norde [10] and surrounding areas on the Caribbean Sea side. Therefore, this weaken precipitation in September probably due to an interaction between local- and large-scale circulations and mechanism. The interactions brings a precipitation decrease in the middle of the rainy season and, as a result, to an intraseasonal variability or bimodal distribution of precipitation known locally as the “Veranito” in Panama or “de San Juan” in other Central America [or, in English, as the “mid-summer drought” [43, 44]].

**Fig 3:**
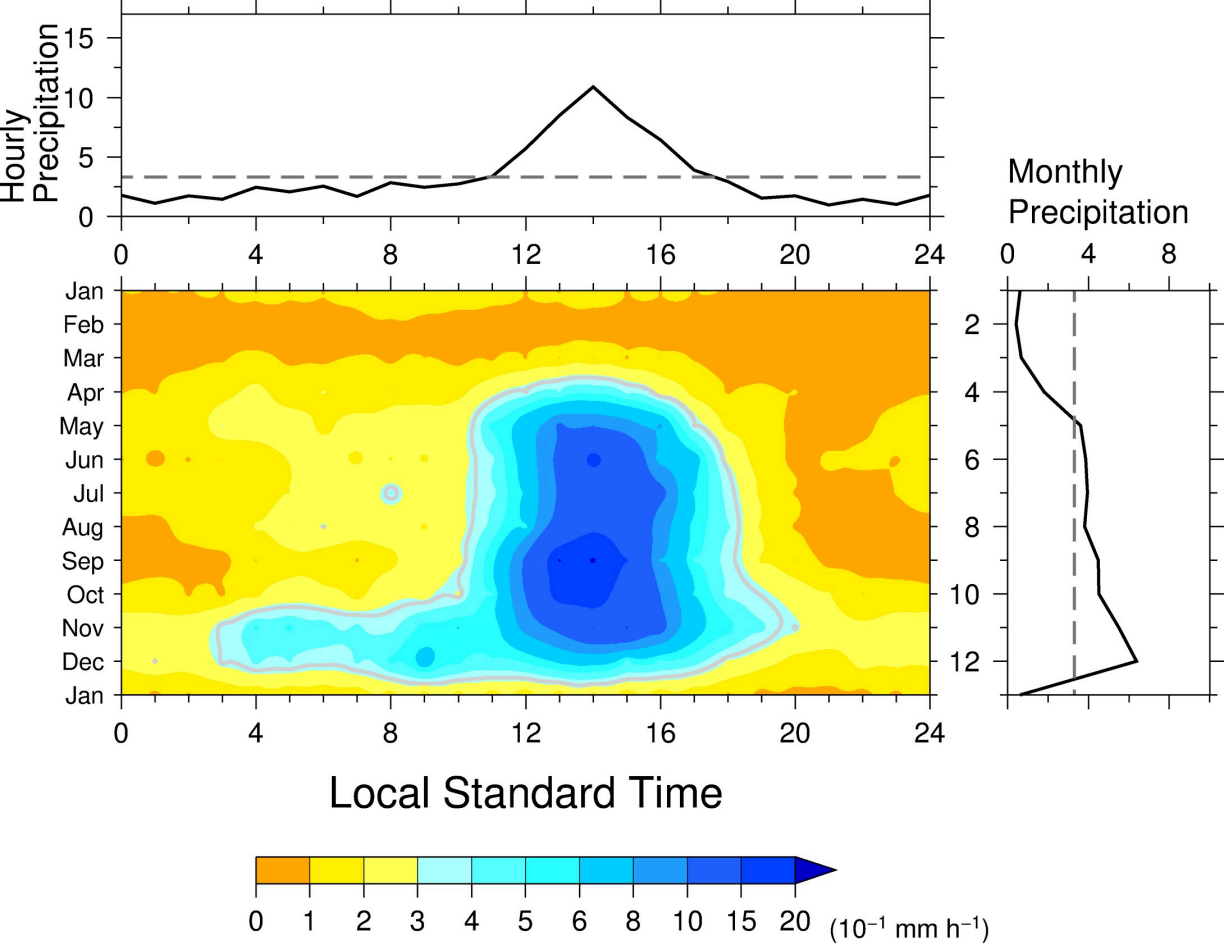
Same as in Fig 2 but for Río Piedras.

At Río Piedras, precipitation showed a diurnal variation similar to that at Chico (Fig 3, center panel), where the climatological annual precipitation was roughly the same (Table 1). Relatively intense precipitation, greater than 0.6 mm h^−1^, occurred from 1300 to 1700 LST from May to November. Hourly precipitation greater than 0.3 mm h^−1^ continued from 0400 to 2100 LST in November and to 1800 LST in December. This late morning precipitation in November was observed at Chico. The magnitude of the climatological annual mean hourly precipitation peak was about two-thirds that at Chico (Fig 3, top panel), but the timing of the peak was the same as in Chico. Climatological monthly precipitation showed seasonality with a peak, in September and December (Fig 3, right panel). A small local minimum occurred in August, when a local maximum was observed at Chico.

**Fig 4:**
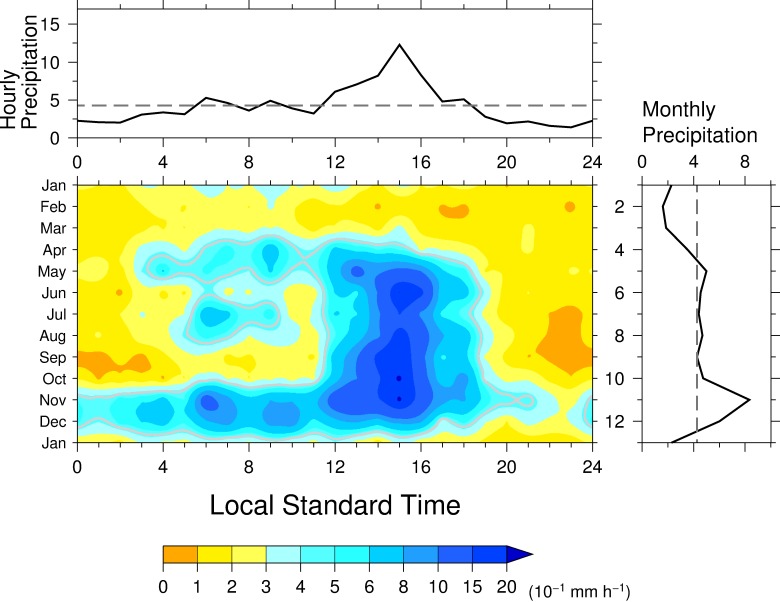
Same as in Fig 2 but for Chamon.

The diurnal variation of precipitation at Chamon varied considerably over the course of the year (Fig 4, center panel). Hourly precipitation greater than 0.3 mm h^−1^ in November and December continued throughout the day except for about 2200 to 2300 LST, and occurred from 0400 to 1200 LST, except in February, March, September, and October. Hourly precipitation greater than 1.5 mm h^−1^ was mostly limited to between 1400 and 1600 LST. The highest peak in the diurnal variation of hourly precipitation occurred at 1500 LST, whereas the second highest peak was at 0600LST. This morning peak, especially that in November and December, is similar to the morning peak that characterizes the oceanic regime [24]. There was more morning precipitation in April than there was in the afternoon. Although the afternoon precipitation was enhanced during the rainy season, the diurnal variation of precipitation at Chamon also showed distinct seasonal changes in the morning. This weaker morning precipitation in June, September, and October occurs in a prolonged mid-summer drought period. A local minimum in the diurnal variation of hourly precipitation at 1100 LST (Fig 4, top panel), during which precipitation intensity did not reach 0.3 mm h^−1^, was observed from June to October. The diurnal variation was very weak in February and March when the monthly mean precipitation was low (Fig 4, right panel), and the variation was bimodal in November, with each peak exceeding 1.5 mm h^−1^. The climatological annual mean hourly precipitation also showed a large peak at 1400 LST, earlier by 1 h than those observed at Chico and Río Piedras, but the earlier low peak was not seen at those two stations (Fig 4, top panel). Climatological monthly precipitation showed a sharp peak in November (Fig 4, right panel).

**Fig 5:**
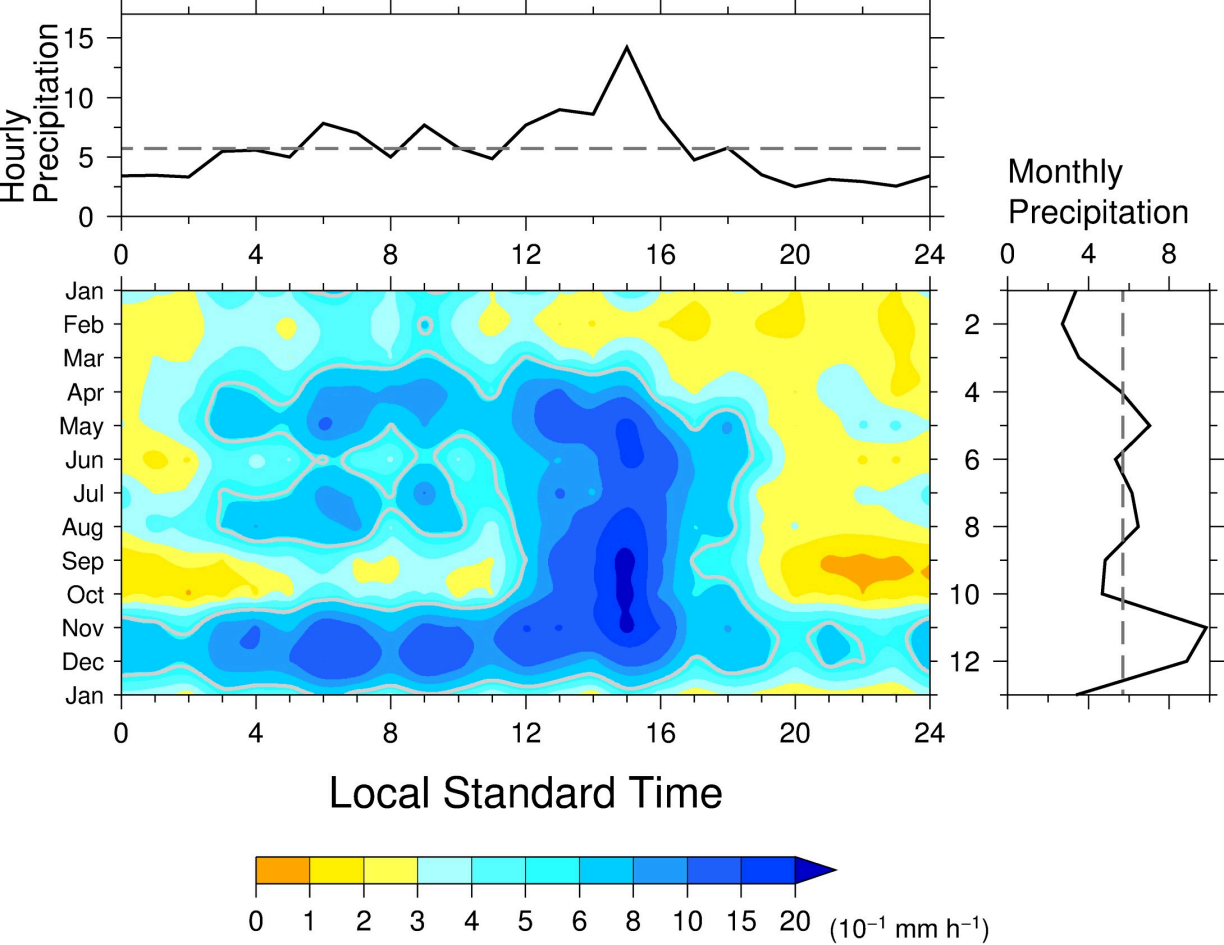
Same as in Fig 2 but for Esperanza.

Hourly precipitation greater than 0.3 mm h^−1^ at Esperanza occurred from 0300 to 1500 LST in January and March but shorten until 1200 LST in February and weakly in the afternoon in those months (Fig 5, center panel). From April to October, hourly precipitation greater than 0.3 mm h^−1^ continued from early morning to evening, although the timing of hourly precipitation in early morning exceeding 0.3 mm h^−1^ became gradually retarded during the season from August to October; however, hourly precipitation less than 0.3 mm h^-1^ in September and October does not occur in the early morning. Intraseasonal weaker morning precipitation are also seen with the same timing of Chamon.

In November and December, hourly precipitation was greater than 0.3 mm h^−1^ throughout the day. Peaks of precipitation exceeding 1.5 mm h^−1^ occurred mostly from 1400 to 1500 LST from May to November. The climatological annual mean hourly precipitation also showed a distinct diurnal variation with a high peak at 1500 LST, as at the other three stations (Fig 5, top panel). In addition, low peaks were observed at 2000 and 2300 LST. The diurnal variation was similar in this respect to that at Chamon but different from the variation at the other two stations. The climatological monthly precipitation was characterized by three distinct peaks (Fig 5, right panel), the only site at which this was the case. The peak in May corresponds to the northward passage of the ITCZ, and the high peak in November, which was also observed at the other stations, corresponds to the southward migration of the ITCZ.

The afternoon peak of the diurnal cycles are fundamentally consistent with those of previous satellite-observation-based studies [20–23]. However, our analysis reveals that there exists a variety of the diurnal cycle even in one-pixel resolution of the satellite observation.

## Discussions

### Data span

The standard span of climatology is 30 years and the 17-yr data record analyzed in this study is short as mentioned in the Data subsection. The available data spans of Chico and Río Piedras are 50 years and 44 years respectively. The results between the 17-yr and full span analyses are compared. The general pattern of the diurnal variation at Chico resembles each other with high pattern correlation coefficient of 0.92 (not shown). Some relative differences between the two analyses against the annual mean precipitation are large at an hour and a month, which often appear in weak precipitation intensity in the dry season. The peaks in both the analyses occurs at 15LST but the peak in the full span analysis is weaker by about 0.25 mm h^-1^ than that of the 17-yr span analysis. The similar results are also found in Rio Piedras. The pattern correlation coefficient at Rio Piedras is 0.93 and the peak is weaker by about 0.3 mm h^-1^. Therefore, the results obtained in this study are climatologically robust.

### Similarity

The similarity of seasonal changes of the diurnal variation between pairs of stations was objectively determined by calculating pattern correlations based on the data shown in the center panels of Figs 2–5 (Table 3, lower triangle). The similarity in the Upper Río Chagres basin was highest between Chico and Río Piedras and between Esperanza and Chamon. The two members of each of pair are relatively close together and at similar elevations. Chico and Río Piedras are located in the downstream part of the Upper Río Chagres basin, at relatively low elevations along major streams. In contrast, Esperanza and Chamon are in the upper part of the basin and at relatively high elevations on topographic highs. The pattern variance is the average of the squared differences of the precipitation intensity from the climatological mean precipitation. The ratios of pattern variances between pairs of stations are all close to 1, except for pairs including Chico, which are close to 2 (Table 3, upper triangle). This result suggests that the afternoon precipitation peak at Chico against the climatological mean precipitation is more remarkable than that of the other three stations. Satellite observations have shown precipitation peak in the early afternoon over land areas of Panama and in the late morning over the Caribbean Sea to the north of Panama [20, 23]. Both late morning and early afternoon precipitation patterns were observed at Chamon and Esperanza, whereas early afternoon precipitation, as in the ground observations, were observed at Chico and Río Piedras. This result suggests that both land and sea precipitation patterns influence the diurnal variation at Chamon and Esperanza, whereas the variation at Chico and Río Piedras is influenced most strongly by the land precipitation pattern. Chamon and Esperanza are located at the highest point in each subbasin of the Upper Chagres River Basin and are exposed to land and sea breezes as seen in other tropical regions (e.g. [29,30]). A persistent flux of moisture from the Caribbean Sea together with orographic lifting can lead to a diurnal variation of precipitation exceeding 0.3 mm h^−1^ in this region in November and December [19]. Land and sea breeze is important to extreme precipitation in the Panama Canal Basin [45].

**Table 3:** (lower triangle) Pattern correlation of seasonal changes of diurnal variations of precipitation between arbitrary pairs of stations. (upper triangle) Ratio of variances between arbitrary pairs of stations, obtained by dividing the variance at each station listed on the left with that of each listed across the top of the table. Lines between Esperanza and Vistamares denote borders of the inside/outside of the Upper Chagres River basin.

Station name	Chico	Río Piedras	Chamon	Esperanza	Vistamares	Dos Bocas	Arca Sonia
Chico		1.65	1.79	1.62	1.38	1.46	0.96
Río Piedras	0.91		1.08	0.98	0.83	0.81	0.58
Chamon	0.87	0.90		0.90	0.77	0.82	0.54
Esperanza	0.76	0.78	0.92		0.85	0.90	0.59
Vistamares	0.94	0.94	0.84	0.70		1.06	0.70
Dos Bocas	0.773	0.83	0.91	0.94	0.75		0.66
Arca Sonia	0.97	0.89	0.84	0.72	0.95	0.75	

Pattern correlation analysis is extended to the other three stations outside the Upper Chagres River Basin. High similarity is identified between the two downstream stations and Vistamares and Arca Sonia to the south of the basin, and between the two upperstream stations and Dos Bocas to the north of the basin. These results suggests that there exists a border separating two regimes of the diurnal variations of precipitation in the Upper Chagres River Basin.

**Fig 6:**
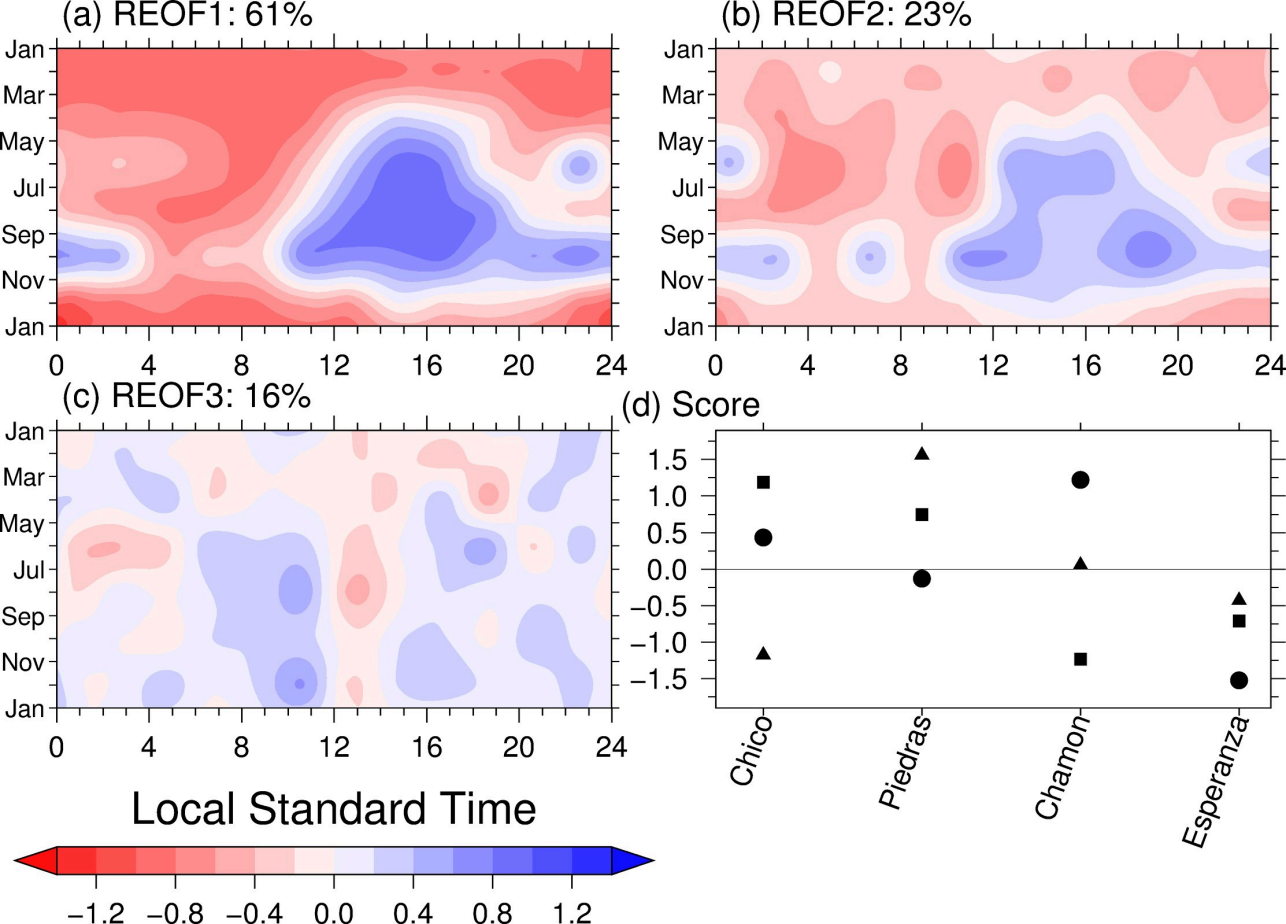
The REOF components of the normalized seasonal changes of the diurnal variation of precipitation at the four stations. (a) First, (b) second, and (c) third modes. The variance explained by each mode is shown at the top of each panel. (d) Principal component scores for the four stations. Squares, circles, and triangles represent the first, second, and third modes respectively.

We extract another aspect of similarities among the 4 stations with REOF analysis [24]. The first mode, REOF1, was characterized by large positive values in the early afternoon from May to November and negative values throughout the day in January to March (Fig 6A). This pattern represents strong diurnal variation with a peak at 1500 LST and positive values almost throughout the day in October. The REOF1 scores were positives for Chico and Río Piedras, indicating that the standardized diurnal range of precipitation at the two stations is stronger than that of the other two stations (Fig 6D). We interpreted REOF1 to include the landside coastal regime [24], in which the precipitation peak occurs in the early afternoon. REOF1 accounted for 61% of the total variance among the four stations. This diurnal variations of solar irradiance primarily induce that of precipitation with land and sea breeze.

The pattern of the second mode of REOF (REOF2; Fig 6B) resembled that of REOF1 but without the peak at 1500 LST and with small values. REOF2 was characterized by large negative values in the morning from January to August and positive values in the early afternoon, especially from 1400 to 1700 LST in June and July, and from 1100 to 1200 and from 1700 to 2000 LST from October to November. The REOF2 scores of Chamon and Esperanza were of opposite sign (Fig 6D). The negative values in the morning in Fig 6B represents features of continuous precipitation from the morning to the evening at Esperanza with a negative large score. The contrast in values between in the morning and the early afternoon shows that of the diurnal variations at Chamon and Esperanza; large diurnal variations at Chamon and small diurnal variations at Esperanza. Esperanza has distinct morning precipitation with early afternoon precipitation while Chamon has distinct early afternoon precipitation only. Orographic precipitation in Dominica in the Tropics lacks in diurnal modulation because the terrain-forced asent [46]. REOF2 represents this lack of the diurnal modulation at Esperanza and indicates the influence of orographic precipitation in the morning.

Large absolute values of the third mode of REOF (REOF3) were characterized by the very limited occurrence of large absolute values greater than 0.4 (Fig 6C). Positive values greater than 0.4 were found locally in late morning and late afternoon from May to December. Distinct large positive values were found at 10 and 11 LST in July, August, November, and December and at 1800 LST in June, while large negative value is found at 13 LST in August. The REOF3 scores distinguish the seasonal changes in the diurnal variation between Chico and Río Piedras (Fig 6D). The absolute values are small and the variance explained by REOF3 is small values of 16%. The pattern of REOF3 does not seem to have a structure reflected by a physical phenomenon, Therefore, REOF3 does not represent a fundamental physical mechanism of the seasonal changes in the diurnal variations of precipitation but rather nonlinear modulation between REOF1 and REOF2, and a local features of Chico and Río Piedras.

We also applied a REOF analysis to the correlation matrix of the seasonal changes of the diurnal variation of precipitation at the seven stations by adding the three stations: Vistamares, Dos Bocas and Arca Sonia.

In summary, the REOF1 analysis divided the four stations between the downstream basin (Chico and Río Piedras) and the upstream basin (Chamon and Esperanza). REOF2 distinguished the two stations in the downstream basin, and REOF3 separated those in the upstream basin. The REOF analysis thus elucidated the graphical similarities and dissimilarities in the normalized seasonal changes of the diurnal precipitation variation and thereby provided insights different from those obtained from the non-normalized graphical patterns (Figs 2–5).

### Near surface winds

**Fig 7:**
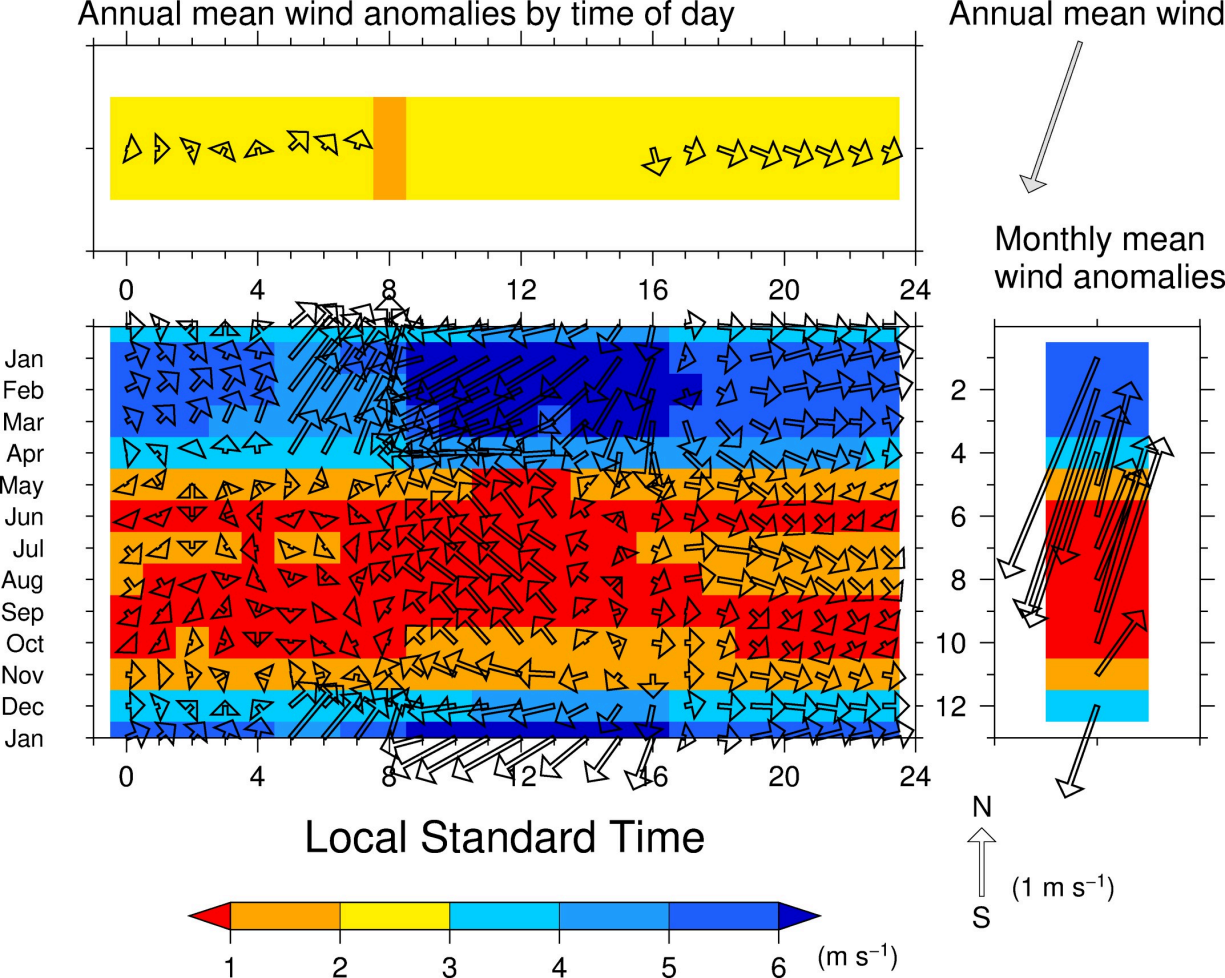
Seasonal changes of (center panel) the diurnal variation of large-scale near-surface wind in each month at the ERA5 grid nearest to the study area, and (right) the monthly mean wind. **(top) Annual mean diurnal variation of wind.** Vectors in the top and center panels denote wind speed and direction anomalies from the climatological annual mean and monthly mean winds, respectively. Vectors in the right panel denote the monthly wind speed and direction anomaly from the climatological annual mean wind. Color shades denote the magnitude of wind speed.

The climatological annual mean large-scale near-surface wind, which is a branch of the Caribbean low-level jet [47], blows from the northeast over the upper Río Chagres basin. The north–south migrations of the ITCZ across Panama are responsible for the seasonal changes in the large-scale near-surface wind (Fig 7, right panel) because the low-level winds blow toward the ITCZ [10]. Large changes in wind direction anomalies observed in May and December correspond to the start and end of afternoon precipitation (Figs 2–5) and to the distribution of high positive REOF1 values (Fig 6).

Seasonal changes of the diurnal variation of the large-scale near-surface wind anomalies relative to the monthly mean wind are shown in the center panel of Fig 7, which represents the large-scale wind field rather than the basin-scale wind field. The diurnal variation of wind during each month is not obvious because the monthly mean wind anomalies showed distinct seasonal changes in wind speed and direction anomalies. From December to April the large-scale near-surface wind anomalies were southwesterly during 0100 to 0700 LST, strong and northeasterly during 0900 to 1600 LST, and westerly during 1700 to 0000 LST. From May to November, the large-scale near-surface wind anomalies were weak during 0000 to 0600 LST, southeasterly during 0700 LST to 1500 LST, and westerly and northwesterly during 1600 to 2300 LST. These seasonal changes in the diurnal variation occurred concurrently with the onset and withdrawal of the rainy season and the passage of the ITCZ. The annual mean near-surface wind anomalies relative to the annual mean wind shows northwesterly to southwesterly during 16 LST to 07 LST and becomes minimal speed during 0800 LST to 15 LST (top panel of Fig 7). When precipitation greater than 0.3 mm h^-1^ during early afternoon generally occurs, annual mean wind blows over the upper Río Chagres basin as an annual mean time scale. The same analysis but with a 6-h temporal resolution of Japanese 55-year Re-Analysis [JRA-55; [48] are performed to clarify the dependence of reanalyses on these features and no distinct differences between the two reanalysis are found.

The diurnal variations of wind at the two ground meteorological stations: Corozal Oeste and Limon Bay: show the land and sea breeze-like temporal pattern are seen (not shown). Land and sea breezes empirically seem to induce afternoon thunderstorms by judging from a geostationary satellite imagery. Therefore, interactions between land and sea breezes and deep convections [21, 22, 31] may be responsible for these complicated variations. These features are lost in the annual mean diurnal variation of the large-scale near-surface wind direction anomalies, relative to the annual mean wind (Fig 7, top panel).

The Caribbean low-level jet shows a seasonal changes of diurnal variations: strongest 925 hPa zonal wind at 0700 LST and meridional wind at 1000 LST for all the seasons, 925 hPa weakest zonal wind at 1900 LST and meridional wind at 1600 LST [49]. It is difficult to quantitatively compare the seasonal changes of the large-scale near-surface wind in Fig 7 with those of the Caribbean low-level jet because the dominant direction components of the large-scale near-surface wind and the jet is meridional and zonal winds respectively. Nonetheless, the dominant direction components shows similar diurnal variations in quality, suggesting the affection of the Caribbean low-level jet on its branch and hence the diurnal variations of precipitation.

### Onset and withdrawal of the rainy season

The seasonal changes of the diurnal variation of precipitation correspond well with the onset and withdrawal of the rainy season occurring with the migration of ITCZ, and “Veranito” [9]. However, there are some discrepancies. The diurnal variations of precipitation at Chico and Chamon in the transitional months, April and December, were not as distinct as those in the other rainy season months. The seasonal changes of the diurnal variation of precipitation were affected by the onset and withdrawal of the rainy season at all stations and at Chamon and Esperanza, also by intraseasonal variability. Sensitivities of convection activities to local-scale complex orography probably vary with each station. These differences in mechanism account for the differences in the seasonal changes of the diurnal precipitation variation within the small area of the upper Río Chagres basin (e.g. Fig 6). In addition to the onset and withdrawal of the rainy season, intraseasonal oscillations such as Madden-Julian Oscillation may influence the complex spatial pattern of the diurnal variations [16, 27, 30] through the local-scale complex orography.

### ENSO

**Fig 8:**
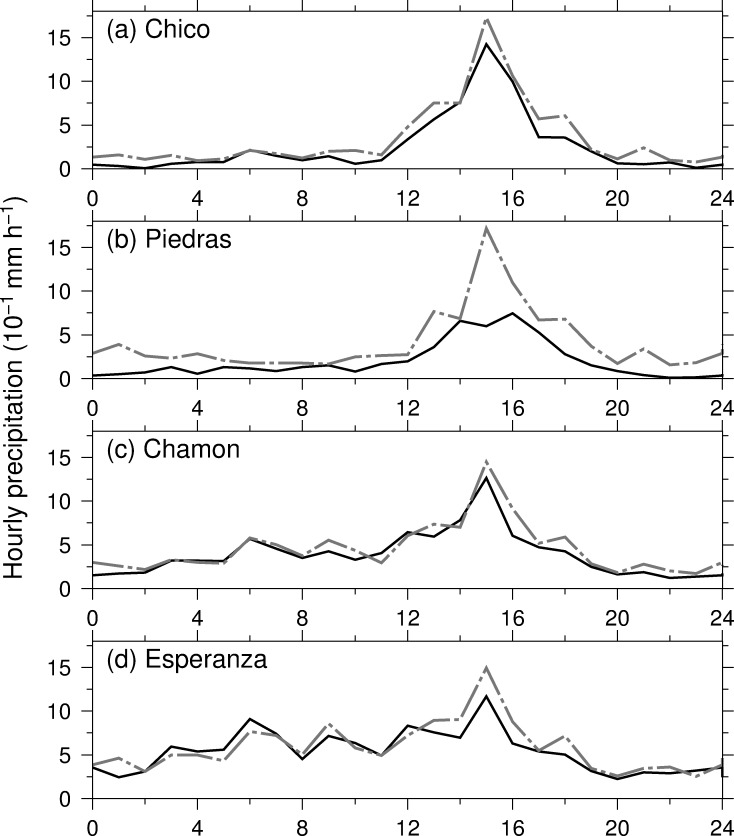
**Annual mean diurnal variation of precipitation during the year at (a) Chico, (b) Rio Piedras, (c) Chamon, and (d) Esperanza stations.** The solid and dashed lines indicate hourly precipitation during the El Niño and La Niña years.

Panama experiences positive and negative precipitation anomalies during the La Niña and El Niño years [13, 50, 51]. Both extreme water deficit and excess have negative influences on Panama Canal navigation, herbivore outbreak, agricultural productions, and others. Here we examine the influence of the ENSO on the diurnal variations of precipitation on annual time-scale. Fig 8 depicts the diurnal variations of precipitation during the El Niño and La Niña years for each station. Hourly precipitation during the La Niña years increase at almost hours at almost stations except for Esperanza, while that of El Niño years decrease so; however, the phases of precipitation peak remain the same at 15 LST during the both years. The differences in hourly precipitation are large in the afternoon when the convection are active and precipitation intensity is strong. Although mechanism of diurnal variations of precipitation remains the same, ENSO events modulate the precipitation intensity as demonstrated in previous studies [16, 26]. This modulation stems from moisture transport for precipitation in either the Caribbean Sea or the eastern tropical Pacific or both [52]. Statistically significant differences in hourly precipitation between during the El Niño and La Niña years are confined in hours and stations due to small numbers of the El Niño and La Niña years and large natural variabilities of hourly precipitation.

### Diurnal variation of streamflow

**Fig 9:**
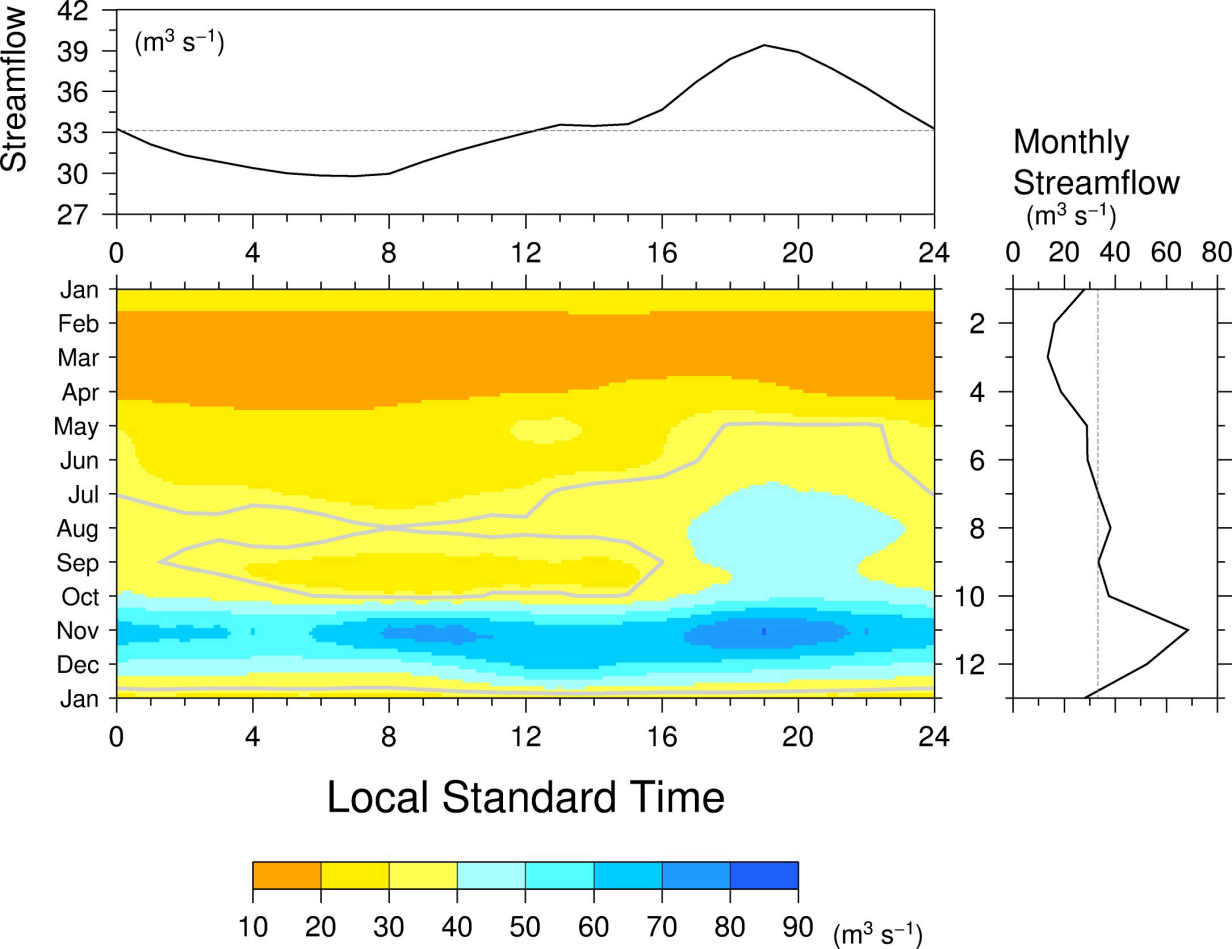
(center) The diurnal variation of streamflow of the Upper Chagres River at Chico station during the year. The gray contours denote annual mean streamflow. (right) Monthly mean streamflow. (top) Annual mean diurnal variation of hourly streamflow. The dashed lines indicate the annual mean hourly (top) and monthly (right) streamflow.

We analyzed diurnal variations of hourly streamflow of the Upper Chagres River at Chico (Fig 9). The afternoon peak occurs at 19LST and seems to start from August to December judging from center panel of Fig 9 but actually starts from April, which is consistent with the start of afternoon precipitation peak seen in Fig 2. Another streamflow peak is identified at 9LST in November, which corresponds to the precipitation peak at 0600 LST. The afternoon precipitation peak leads the afternoon streamflow peak by 4 h, while the morning precipitation peak leads the morning streamflow peak by 3 h. Top panel of Fig 9 shows the annual mean diurnal variations of streamflow. The afternoon streamflow peak is seen as annual mean at 1900 LST and the morning minimum is seen at 0700 LST. The peak of climatological annual mean hourly streamflow is only 30% of the annual mean streamflow, while the peak of climatological mean hourly precipitation exceeds 500% of the annual mean precipitation.

The lag time between precipitation and streamflow peaks and the reduction in peak from precipitation to streamflow are because of hydrological processes: infiltration into soils, subsurface runoff, and river routing processes in the Chico River Basin in quality. These indicate that the diurnal variations of precipitation distinctly affect those of streamflow in the Chico River Basin and that the hydrological processes on the basin modulate both the phase and the magnitude of the peak of the streamflow. The lag time in the Boqueron River basin with the area of 91 km^2^ also flowing to the Lago Alhajuela is 1–3 h [52] is qualitatively consistent with the lag time of the upper Río Chagres basin.

## Concluding summary

This study demonstrated that the seasonal changes of the diurnal variation of precipitation show spatial variability among four rain gauge stations in the upper Río Chagres basin, Panama, despite the small area of the basin. At Chico and Río Piedras, the seasonal changes were similar, with an early afternoon precipitation peak at 1500 LST, but Río Piedras also exhibited late morning precipitation in November and December. The diurnal variation of precipitation at Chamon and Esperanza varied from month to month. In November and December, in addition to the early afternoon precipitation, hourly precipitation always exceeded 0.3 mm h^−1^, except at 2200 LST in December at Chamon. At Esperanza, morning precipitation peaks in January and March distinguished the seasonal changes of the diurnal variation of precipitation there from those at the other stations.

We quantified the similarities among the four stations by pattern correlation analysis of the seasonal changes of the diurnal variation of precipitation. The results grouped the stations into two pairs: Chico and Río Piedras, and Chamon and Esperanza. The two stations in each pair are geographically close: Both Chico and Río Piedras are located in the downstream basin, whereas Chamon and Esperanza are located in the upstream basin. REOF analysis confirmed these similarities and further decomposed the data into three components: early afternoon precipitation in the rainy season, morning precipitation from January to August, and late morning and afternoon precipitation from May to December.

Seasonal changes of the diurnal variation of large-scale near-surface wind were small compared to the seasonal changes of monthly mean large-scale near-surface wind associated with the migration of the ITCZ. Small-scale winds such as land and sea breezes may influence the diurnal variation of precipitation. The onset and withdrawal of the rainy season primarily account for the seasonal changes of the diurnal variation of precipitation, but they are modulated by intraseasonal variabilities in precipitation, such as the Veranito.

The present study showed that large spatial variability exists in the seasonal changes of the diurnal variation of precipitation in the upper Río Chagres basin and that the changes are associated with the onset and withdrawal of the rainy season. However, owing to limited data availability, physical processes are not explained in the present study. Orographic precipitation [8], interaction between land and sea breezes and deep convection [21], intraseasonal variabilities in precipitation due to Madden-Julian oscillations [16], and other factors may produce the spatial variability in the seasonal changes of the diurnal variations of precipitation in this small basin (414 km^2^). These factors should be examined in future studies.

Diurnal variations of precipitation in the tropical region have been investigated using the satellite data [e.g., 1, 21, 23, 24, 26] due to low observation availability of hourly precipitation with long-term high quality. This study may provide ground-based validation of the seasonal changes of the diurnal variations of hourly precipitation for satellite-based precipitation products. Moreover, the spatial variability in the seasonal changes pf the diurnal variations of precipitation exists in a footprint similar to that of the satellite-based precipitation products. Both the satellite-based and ground-based observations are essential to further understand the seasonal changes of the diurnal variations of precipitation and to develop improved satellite-based precipitation products with high spatio-temporal resolution.

Numerical experiments performed with a non-hydrostatic regional climate model with a high horizontal resolution would help us to understand the large spatial variability in the seasonal changes of the diurnal variation of precipitation in small basins such as this one. A regional climate model with a 2-km horizontal resolution could simulate seasonal changes of the diurnal variation of precipitation in Costa Rica [17]. Where there is large variability and steep topography, a horizontal model resolution of 1 km or even 500 m may be necessary to simulate seasonal changes in the diurnal variation of precipitation in a small basin. It is a scientific interest how a future climate under increasing greenhouse gas concentrations will affect the diurnal variations as many studies reported many impacts of a changing climate on the climates in Panama [53, 54, 55]. We cannot investigate this question without the numerical experiments as well.

## Supporting information

S1 Dataset Time series of hourly precipitation at the eight stations listed in Table 1. (ZIP)

## References

[1] Garreaud R, Wallace JM. The diurnal march of convective cloudiness over the Americas. Mon Wea Rev. 1997;125:3157–3171. 10.1175/1520-0493(1997)125<3157:TDMOCC>2.0.CO;2

[2] Anderson BT, Kanamaru H. The diurnal cycle of the summertime atmospheric hydrologic cycle over the southwestern United States. J Hydrometeorol. 2005;6:219–228. 10.1175/JHM413.1

[3] Lundquist JD, Cayan DR. Seasonal and spatial patterns in diurnal cycles in streamflow in the western United States. J Hydrometeorol. 2002;3: 591–603. 10.1175/1525-7541(2002)003<0591:SASPID>2.0.CO;2

[4] Ibrahimi MK, Miyazaki T, Nishimura T. A high measurement frequency based assessment of shallow groundwater fluctuations in Metouia Oasis, South Tunisia. Hydrol Res Lett. 2010;4:75–79.

[5] Chikita K, Okumura Y. Dynamics of turbidity currents measured in Katsurazawa Reservoir, Hokkaido, Japan. J Hydrol. 1990;117:323–338. 10.1016/0022-1694(90)90099-J

[6] Makita N, Kosugi Y, Sakabe A, Kanazawa A, Ohkubo S, Tani M. Seasonal and diurnal patterns of soil respiration in an evergreen coniferous forest: Evidence from six years of observation with automatic chambers. PloS ONE, e0192622 10.1371/journal.pone.0192622 29432465 PMC5809067

[7] Hastenrath S. On modes of tropical circulation and climate anomalies. J Atmos Sci. 1978;35:2222–2231.

[8] Nakaegawa T, Kitoh A, Murakami H, Kusunoki S. Annual maximum 5-day rainfall total and maximum number of consecutive dry days over Central America and the Caribbean in the late 21st century projected by an atmospheric general circulation model with three different horizontal resolutions. Theorcal Appl Climatol. 2014b;16:155–168. 10.1007/s00704-013-0934-9

[9] UNESCO, 2008. Balance hídrico superficial de Panamá, período 1971–2002. Documento Técnicos del PHI-LAC 9, 133pp. Available from: http://unesdoc.unesco.org/images/0015/001591/159103s.pdf [Accessed 26 September 2019]

[10] Nakaegawa T, Arakawa O, Kamiguchi K. Investigation of climatological onset and withdrawal of the rainy season in Panama based on a daily gridded precipitation dataset with a high horizontal resolution. J Clim. 2015;28:2745–2763. 10.1175/JCLI-D-14-00243.1

[11] Mitchell T, Wallace J. The Annual Cycle in Equatorial Convection and Sea Surface Temperature. J Clim. 1992;5(10):1140–1156. 10.1175/1520-0442(1993)006<1678:C>2.0.CO;2

[12] Durán-Quesada AM, Revoita M, Gimeno L. Precipitation in tropical America and the associated sources of moisture: a short review, Hydrol Sci J. 2012;57(4):612–624, 10.1080/02626667.2012.673723

[13] Leigh EG, Windsor DM, Rand AS, Foster RB. The impact of the “El Niño” drought of 1982–83 on a Panamanian semi deciduous forest. Elsevier Oceanogr seri. 1990;52:473–486.

[14] Hastenrath S. The intertropical convergence zone of the eastern Pacific revisited. Int J Climatol. 2002;22:347–356. 10.1002/joc.739

[15] Schultz DM, Bracken WE, Bosart LF. Planetary- and synoptic-scale signatures associated with Central American cold surges. Mon Wea Rev. 1998;126:5–27. 10.1175/1520-0493(1998)126<0005:PASSSA>2.0.CO;2

[16] Poveta G, Mesa OJ, Salazar LF, Arias PA, Moreno HA, Vieira SC, et al The diurnal cycle of precipitation in the Tropical Andes of Colombia. Mon Wea Rev. 2005;133:228–240. 10.1175/MWR-2853.1

[17] Amador JA, Saenz F. Diurnal cycle on the Caribbean slope of Costa Rica: An observational and numerical study. A21B-06. In Meeting of the Americas, Cancun, Mexico. 14–17 May 2013. Available from: http://moa.agu.org/2013/eposters/eposter/a21b-06/ [Accessed 26 September 2019].

[18] Oki T, Musiake K. Seasonal change of the diurnal cycle of precipitation over Japan and Malaysia. J Appl Meteorol Climatol. 1994;33:1445–1463. 10.1175/1520-0450(1994)033<1445:SCOTDC>2.0.CO;2

[19] Rapp AD, Peterson AG, Frauenfeld OW, Quiring SM, Roark EB. 2014. Climatology of storm characteristics in Costa Rica using the TRMM Precipitation Radar. J Hydrometeorol. 2014;15:2615–2633. 10.1175/JHM-D-13-0174.1

[20] Mapes BE, Warner TT, Xu M, Negri AJ. Diurnal patterns of rainfall in Northwestern South America. Part I: Observations and Context. Mon Wea Rev. 2003;131:799–812. 10.1175/1520-0493(2003)131<0799:DPORIN>2.0.CO;2

[21] Biasutti M, Yuter SE, Burleyson CD, Sobel AH. Very high resolution rainfall patterns measured by TRMM precipitation radar: seasonal and diurnal cycles. Clim Dyn. 2012;39:239–258.

[22] Negri AJ, Xu L, Adler RF. A TRMM-calibrated infrared rainfall algorithm applied over Brazil. J Geophy Res. 2002;107:8048 10.1029/2000JD000265

[23] Sorooshian S, Gao X, Hsu K, Maddox RA, Hong Y, Gupta HV, Imam B. Diurnal variability of tropical rainfall retrieved from combined GOES and TRMM satellite information. J Clim. 2002;15:983–1001.

[24] Kikuchi K, Wang B. Diurnal precipitation regimes in the global tropics. J Clim. 2008;21:2680–2696. 10.1175/2007JCLI2051.1

[25] Jiang Q. 2003 Moist dynamics and orographic precipitation. Tellus A: Dyn Meteorol Oceanogr. 55.4: 301–316.

[26] Qian JH, Robertson AW, Moron V. 2010 Interactions among ENSO, the monsoon, and diurnal cycle in rainfall variability over Java, Indonesia. J Atmos Sci. 67: 3509–3524. 10.1175/2010JAS3348.1

[27] Perdigón-Morales J, Romero-Centeno R, Barrett BS, Ordoñez P. Intraseasonal variability of summer precipitation in Mexico: MJO influence on the midsummer drought. J Clim, 2019: 10.1175/JCLI-D-18-0437.1

[28] Harmon RS. 2005. An Introduction to the Panama Canal Watershed In: Harmon R. S. ed. The Río Chagres, Panama, Water Science and Technology Library, Springer 2005;52:19–28.

[29] Knox RG, Ogden FL, Dinku T. 2005. Using TRMM to Explore Rainfall Variability in the Upper Río Chagres Catchment, Panama In: Harmon R. S., ed. The Río Chagres, Panama, Water Science and Technology Library, Springer 2005;52:211–226.

[30] Hsu HH, Lee MY. Topographic effects on the eastward propagation and initiation of the Madden–Julian oscillation. J Clim, 2005;18:795–809. 10.1175/JCLI-3292.1

[31] Bhatt BC, Nakamura K. A climatological‐dynamical analysis associated with precipitation around the southern part of the Himalayas. J Geoph. Res.: Atmos. 2006;111:D02115 10.1029/2005JD006197

[32] Zhou L, Wang Y. Tropical Rainfall Measuring Mission observation and regional model study of precipitation diurnal cycle in the New Guinean region. J Geophy Res. 2006;111:D17104 10.1029/2006JD007243

[33] Fábrega J, Nakaegawa T, Pinzón R, Nakayama K, Arakawa O. SOUSEI Theme-C modeling group. Hydroclimate projections for Panama in the 21st Century. Hydrol Res Lett. 2013;7:23–29. 10.3178/hrl.7.23

[34] Göktürk OM, Bozkurt D, Şen Ö.L, Karaca M. Quality control and homogeneity of Turkish precipitation data. Hydrol Proces. 2008: 22: 3210–3218.

[35] Wang XL, Feng Y. 2013. RHtestsV4 User Manual. Climate Research Division, Atmospheric Science and Technology Directorate, Science and Technology Branch, Environment Canada, Toronto, Ontario, Canada. Available from: http://etccdi.pacificclimate.org/RHtest/RHtestsV4_UserManual_10Dec2014.pdf [Accessed 26 September 2019]

[36] Wang XL, 2008a Accounting for autocorrelation in detecting mean-shifts in climate data series using the penalized maximal t or F test. J Appl Meteor Climatol. 47, 2423–2444.

[37] Wang XL., 2008b Penalized maximal F-test for detecting undocumented mean-shifts without trend-change. J Atmos Ocea Tech, 25 (No. 3), 368–384. 10.1175/2007/JTECHA982.1

[38] Wang B, LinHo. Rainy season of the Asian–Pacific summer monsoon. J Clim, 2002: 15, 386–398, 10.1175/1520-0442(2002)015,0386:RSOTAP.2.0.CO;2

[39] Alfaro EJ. Some characteristics of the annual precipitation cycle in Central America and their relationships with its surrounding tropical oceans. Tóp Meteor Oceanogr,2002:9,88–103

[40] IMD, 1943: Climatological atlas for airmen. India Meteorological Department, 100 pp.

[41] Hersbach H, Dee D. ERA5 reanalysis is in production. ECMWF newsletter 2016: 147.7.

[42] Ishizaki Y, Nakaegawa T, Takayabu I. Validation of precipitation over Japan during 1985–2004 simulated by three regional climate models and two multi-model ensemble means. Clim Dyn. 2012;39:185–206. 10.1007/s00382-012-1304-5

[43] Magaña V, Amador JA, Medina S. The mid-summer drought over Mexico and Central America. J Clim. 1999;12:1577–1588. 10.1175/1520-0442(1999)012<1577:TMDOMA>2.0.CO;2

[44] Perdigón-Morales J, Romero-Centeno R, Ordoñez P, Barrett BS. 2018 The midsummer drought in Mexico: perspectives on duration and intensity from the CHIRPS precipitation database. Int J Climatol. 38 (5): 2174–2186.

[45] Wang J, Shamir E, Georgakakos KP. Study of extreme precipitation over the Panama Canal Watershed. Hydrologic Research Center, Tech. Rep. 2007;6:158pp. Available from: https://www.hrcwater.org/hrc-technical-reports/ [Accessed 26 September 2019]

[46] Smith R.B., Schafer P., Kirshbaum D.J. and Regina E., 2009 Orographic precipitation in the tropics: Experiments in Dominica. JAtmos Sci, 66(6), pp.1698–1716.

[47] Nakaegawa T, Kitoh A, Ishizaki Y, Kusunoki S, Murakami H. Caribbean low-level jets and accompanying moisture fluxes in a global warming climate projected with CMIP3 multi-model ensemble and fine-mesh atmospheric general circulation models. Int JClimatology. 2014a; 34:964–977. 10.1002/joc.3733

[48] Kobayashi S, et al The JRA-55 Reanalysis: General specifications and basic characteristics. J Meteorol SocJp. 2015;93:5–48. 10.2151/jmsj.2015-001

[49] Cook KH, Vizy EK, 2010 Hydrodynamics of the Caribbean low-level jet and its relationship to precipitation. J Clim. 23(6), pp.1477–1494. 10.1175/2009JCLI3210.1.649

[50] Lachniet MS, et al A 1500‐year El Niño/Southern Oscillation and rainfall history for the isthmus of Panama from speleothem calcite. J Geophyp-sical Res: Atmos, 2004, 109.D20. 10.1029/2004JD004694

[51] Durán-Quesada AM, Gimeno L, Amador J. Role of moisture transport for Central American precipitation. Earth System Dynamics, 2017:8(1): 147–161.

[52] Reynolds JE, et al Definitions of climatological and discharge days: do they matter in hydrological modelling?, Hydrol Sci Journal, 2018;63(5):836–844. 10.1080/02626667.2018.1451646 doi: 10.5194/esd-8-147-2017

[53] Pinzon R, Hibino K., Takayabu I, Nakaegawa T. Virtual experiencing future climate changes in Central America with MRI-AGCM: climate analogues study. Hydrolal ResLetters. 2017;11:106–113. 10.3178/hrl.11.107

[54] Nakaegawa T, Kitoh A, Kusunoki S, Murakami H, Arakawa O. Hydroclimate change over Central America and the Caribbean in a global warming climate projected with 20-km and 60-km mesh MRI atmospheric general circulation models Pap Meteorol Geoph. 2014:65: 15–33.

[55] Kusunoki S, Nakaegawa T, Pinzon R, Sanchez J, Fabrega JR. Future precipitation changes over Panama projected with the atmospheric global model MRI-AGCM3.2. Clim Dyn. 2019: 11–16.

