# Supplementary material for: Seasonal changes of the diurnal variation of precipitation in the upper Río Chagres basin, Panamá
Source: PLoS One. 2019 Dec 16;14(12):e0224662. doi: 10.1371/journal.pone.0224662 (PMC6913943; doi:10.1371/journal.pone.0224662)
Supplement: S1 Dataset — (ZIP) [file pone.0224662.s001.zip › PLOSONE/readme20191124.docx]

---------------------------------------------------------------------

DATASET OVERVIEW

Dataset Working Title:

Hourly precipitation data in the Upper Chagres River Basin in the Panama Canal Catchment, Panama

Investigators: (full name as formatted for manuscripts, email address, ORCID ID)

Tosiyuki Nakaegawa, [tnakaega@mri-jma.go.jp](mailto:tnakaega@mri-jma.go.jp), 0000-0003-4821-4190

Point of Contact: (name, email, institution)

Hector A. De Lima, [HADeLima@pancanal.com](mailto:HADeLima@pancanal.com), Autoridad del Canal de Panamá

Summary: (overview describing the variables in the dataset and how they were derived)

Hourly precipitation data at seven rain gauge stations: Chico, Río Piedras, Chamon, and Esperanza in the Upper Chagres River Basin, and Vistamares, Dos Bocas, and Arca Sonia in the Panama Canal Catchment.

Keywords:

precipitation; hourly; diurnal variation; Upper Chico River basin; Panama; Panama Canal

Acknowledgments: (include funding sources)

The hourly precipitation data were operationally collected by the Autoridad del Canal de Panamá.

Related Publication: (include DOIs)

Tosiyuki Nakaegawa, Reinhardt Pinzón, Jose Fábrega, Johnny A. Cuevas, Hector A. De Lima, Eric Cordoba, Keisuke Nakayama, Josue Ivan Batista Lao, Alcely Lau Melo, Diego Arturo Gonzalez, and Shoji Kusunoki. 2019. Seasonal changes of the diurnal variation of precipitation in the upper Río Chagres Basin, Panama. PLOS ONE

Related Datasets: (include DOIs)

N/A

---------------------------------------------------------------------

DATASET CHARACTERISTICS

Spatial Resolution: (e.g., 1000 m, 2 degrees, point locations): point locations

Temporal Resolution: (e.g., daily, annual, one-time estimate) hourly

Temporal Coverage: (e.g., YYYY-MM-DD to YYYY-MM-DD): mostly 2000-2016

File Naming Convention: XXX-hourly.csv

The above 4 dataset characteristics are listed in the table below.

| Station name | Longitude  (W) | Latitude  (N) | Elevation  (m) | Naming Convention | period |
| --- | --- | --- | --- | --- | --- |
| Chico | 79° 30' 35" | 9° 15' 49" | 104 | CHI | 2000-2016 |
| Río Piedras | 79° 23' 52" | 9° 16' 52" | 198 | RPA | 2000-2011 |
|  | 79° 23' 58" | 9° 16' 55" | 201 | RPA | 2011-2016 |
| Chamon | 79° 19' 06" | 9° 20' 31" | 2100 | CHM | 2000-2016 |
| Esperanza | 79° 21' 08" | 9° 24' 35" | 1780 | EZA | 2000-2016 |
| Vistamares | 79° 24' 05" | 9° 14' 04" | 969 | VTM | 2000-2016 |
| Dos Bocas | 79° 25' 52" | 9° 27' 09" | 250 | DBK | 2000-2016 |
| Arca Sonia | 79° 30' 54" | 9° 11' 36" | 261 | ARC | 2000-2016 |

Projection Information: N/A

File Format and Number: CSV

File Descriptions: (file names, units, and descriptions) file names: XXX-hourly.csv; units: Mm hr ^-1;

Data Dictionary: (variables, units, and descriptions that define jargon, acronyms, abbreviations, etc.)

First line: Station name and its abbreviations in the above table : e.g. Arca Sonia (ARC),

Second line: Date,Precip (mm) (fixed)

Third line and after: e.g. MM/DD/YYYY HH:00, precipitation: e.g. 4/29/1999 12:00,2.54

---------------------------------------------------------------------

APPLICATION & DERIVATION

Hourly precipitation data in tropical (rainforest) region were invaluable for meteorological and climatological study.

(What is the significance and application of the data?)

---------------------------------------------------------------------

QUALITY ASSESSMENT

The raw 5-min data are quality controlled with a commercial software (AQUARIUS Time-Series, version 3.7; https://aquaticinformatics.com/; © Copyright, 2019 Aquatic Informatics Inc.)

Estimate of Uncertainty: (how data quality was assessed)

---------------------------------------------------------------------

DATA ACQUISITION, MATERIALS & METHODS

(Include enough information so a user can determine if the data are usable for their application. If applicable, begin with a description of the study sites. Images are permitted. Please provide a description of the image.)

Platforms: (e.g., DC-8, Aqua, Environmental Modeling, Field Investigation)

Instruments: (e.g., PALSAR, LIDAR, computer) The rain gauges used in these stations are tipping bucket type one (Model 5050P; HydroLynx Systems, Inc.)

---------------------------------------------------------------------

REFERENCES

Same as in Related Articles above

(Provide a bibliography, including DOIs, for any literature or data products/services that are referenced in this document.)

---------------------------------------------------------------------

BROWSE IMAGE

(Data providers are encourged to include an image or recommend a data file from which to create an image that describes the dataset.)

Image File Name: N/A

Image Description: N/A

---------------------------------------------------------------------

SUPPLEMENTAL FILES

(Data providers may include ancillary files to accompany the main dataset. These include supplemental tables, color tables, XML/KMZ files, photos, reports, etc.)

Companion File Name: N/A

Companion Description: N/A

---------------------------------------------------------------------
